# Case reports describing treatments in the emergency medicine literature: missing and misleading information

**DOI:** 10.1186/1471-227X-9-10

**Published:** 2009-06-15

**Authors:** Tiffany P Richason, Stephen M Paulson, Steven R Lowenstein, Kennon J Heard

**Affiliations:** 1 University of Colorado School of Medicine, Aurora CO, USA; 2 Denver Health Residency in Emergency Medicine, Denver Health, Denver CO, USA; 3 The Colorado Emergency Medicine Research Center, Division of Emergency Medicine, Department of Surgery, University of Colorado School Denver of Medicine, Aurora CO, USA; 4 Rocky Mountain Poison and Drug Center, Denver Health, Denver CO, USA

## Abstract

**Background:**

Although randomized trials and systematic reviews provide the "best evidence" for guiding medical practice, many emergency medicine journals still publish case reports (CRs). The quality of the reporting in these publications has not been assessed.

**Objectives:**

In this study we sought to determine the proportion of treatment-related case reports that adequately reported information about the patient, disease, interventions, co-interventions, outcomes and other critical information.

**Methods:**

We identified CRs published in 4 emergency medicine journals in 2000–2005 and categorized them according to their purpose (disease description, overdose or adverse drug reactioin, diagnostic test or treatment effect). Treatment-related CRs were reviewed for the presence or absence of 11 reporting elements.

**Results:**

All told, 1,316 CRs were identified; of these, 85 (6.5%; 95CI = 66, 84) were about medical or surgical treatments. Most contained adequate descriptions of the patient (99%; 95CI = 95, 100), the stage and severity of the patient's disease (88%; 95CI = 79, 93), the intervention (80%; 95CI = 70, 87) and the outcomes of treatment (90%; 95CI = 82, 95). Fewer CRs reported the patient's co-morbidities (45%; 95CI = 35, 56), concurrent medications (30%; 95CI = 21, 40) or co-interventions (57%; 95CI = 46, 67) or mentioned any possible treatment side-effects (33%; 95CI = 24, 44). Only 37% (95CI = 19, 38) discussed alternative explanations for favorable outcomes. Generalizability of treatment effects to other patients was mentioned in only 29% (95CI = 20, 39). Just 2 CRs (2.3%; 95CI = 1, 8) reported a 'denominator" (number of patients subjected to the same intervention, whether or not successful.

**Conclusion:**

Treatment-related CRs in emergency medicine journals often omit critical details about treatments, co-interventions, outcomes, generalizability, causality and denominators. As a result, the information may be misleading to providers, and the clinical applications may be detrimental to patient care.

## Background

*An air of serving the common good clings to the process of reporting as general information the results of one's own extensive experience*. [1]

For almost 200 years, clinical case reports have been a prominent feature of medical journalism. Penicillin, ether and insulin were first introduced in case reports or case series. [1] The clinical manifestations of AIDS were first described in case reports,[2] and in 1981 a single case report was the basis for the hypothesis that oral contraceptives increased the risk of venous thromboembolic disease. [3] When the *Journal of the American Medical Association *assembled a collection of fifty-one landmark articles in medicine, five (10 percent) were case reports. [4]

Today, MEDLINE lists more than one million case reports, and this number increases at a rate of 40,000 per year. [5] In January, 2007 the first peer-reviewed journal dedicated specifically to case reports, *The Journal of Medical Case Reports*, was introduced. [6]

At the same time, there is continuing debate about the validity of case reports and their value to practicing clinicians. Some case reports have proved to be poor guides to medical practice. Gastric freezing for bleeding ulcers,[7] intravenous verapamil for ventricular tachycardia, [8] physostigmine for tricyclic anti-depressant poisoning[9] and MAST suit inflation for multi-system trauma[10] began with misleading, misunderstood or misapplied case reports. Indeed, according to Moses, "nearly every discarded, once-popular therapy was probably supported by a series of favorable cases." [1]

In the emergency medicine literature, one-fourth of all publications are case reports,[11] but little is known about their quality. Therefore, we conducted this review oftreatment-related case reports from thethe emergency medicine literature. We had two specific aims: First, to determine how often treatment-related case reports included critical information about the patient, disease, co-morbidities, interventions, co-interventionsand outcomes; and second, to measure the frequency with which emergency medicine case reports included a meaningful discussion of the generalizability of their results and alternative explanations for their favorable outcomes.

## Methods

### Theoretical Model

Case reports are unstructured anecdotes that sit at the bottom of the "hierarchy" of medical evidence. [12] While there are standardized reporting requirements for systematic reviews[13,14] and for studies of treatment,[15] diagnosis[16] and cost-effectiveness,[17] there are no accepted guidelines for the reporting of clinical case reports. Nonetheless, a review of standard textbooks of clinical epidemiology and medical evidence users' guides suggests a number of basic elements that should be routinely reported. A case report should present enough information to enable a clinician-reader to understand the nature, stage and severity of the patient's disease, the treatments rendered and the outcomes that were measured. It should also be the author's responsibility to outline important limitations to the generalizability of their case report. In addition, authors should acknowledge that any inference that the treatment *caused *the observed outcome is, at best, tentative they should enumerate the competing hypotheses (for example, the natural resolution, co-interventions, spontaneous variability of a patient's signs or symptoms) that could have contributed to the favorable outcome. [18]

### Study design

This was a retrospective, descriptive review of treatment-related case reports published in the emergency medicine literature.

### Selection of studies

All case reports from four prominent English-language emergency medicine journals (*Annals of Emergency Medicine*, *Academic Emergency Medicine*, *Journal of Emergency Medicine *and *American Journal of Emergency Medicine*) published between 2000–2005 were identified and retrieved via an Ovid electronic search of MEDLINE, using the limit "case report." Abstracts were reviewed and the reports were classified as having one of four purposes: novel presentation of a disease; adverse drug reaction; utility of a diagnostic test; or description of a treatment effect. Only treatment-related case reports were selected for detailed review. A "treatment-related case report" was defined as a report that described a medical or surgical intervention that altered, or failed to alter, the course of a patient's illness.

A "case report" was defined as a detailed presentation of a single case or a small number of cases. When more than one case was presented, it was accepted as a case report only if individual, patient-specific information (age and gender, disease description, interventions and outcomes) was reported; if this information was provided in aggregated form (for example, means or proportions) the publication was considered a descriptive research report and was excluded. Where more than one case was presented, only the first case was reviewed, in order to avoid over-representation of a single author.

### Measurements

Each case report was analyzed independently by the senior authors (both experienced clinician-scientists) for the presence or absence of 11 elements listed in Table 1. These elements were selected after a review of standard textbooks of clinical epidemiology,[19,20] guidelines for critical appraisal of studies of treatments and harms[21] and the Users' Guides to Evidence-Based Medicine. [12]

**Table 1:** Critical Reporting Elements for Case Reports

**Description of Patient and Disease**	
**Age and gender**	Patient age and gender are provided

**Definition, stage and severity of disease**	Description of patient and his illness prior to intervention are clearly described. Criteria utilized to confirm patient's disease are clear (scoring criteria, imaging, laboratory studies, pathologic specimens).

**Co-morbid conditions**	Relevant co-morbid conditions listed as present or absent

**Patient medications**	Relevant medications listed or stated that none were present

**Description of Interventions**	

**Route and dose of medications or details of procedure**	Route and dose of medications stated clearly; or, details of procedure provided.

**Co-interventions**	Expected or unexpected co-interventions listed, or statement that none were administered.

**Description of Outcomes**	

**Clinically relevant outcomes defined**	Critical outcome measures are clearly defined. The authors state the time period when outcomes were assessed, the durability of observed outcomes, their importance to patients and whether other relevant outcomes were or were not measured.

**Side effects reported**	All side effects clearly described, or stated that none were observed.

**Threats to Generalizability and Validity**	

**Generalizability**	The report highlights patient attributes, disease characteristics, expertise of clinician-authors, treatment setting or other factors that might limit generalizability or applicability of the treatment to other settings or patient populations.

**Alternative explanations**	Authors describe alternative explanations for the observed treatment effect, such as the natural resolution of the symptoms or signs, spontaneous variation, co-interventions or observer bias.

**Information about "denominator"**	There is a clear reference to the number of other patients who received this intervention, whether successful or not.

For each element, a rating of "present" or "absent" was assigned. Credit was given if the author mentioned the element, whether or not specific details were provided. For example, a case report that stated, *The patient tolerated the treatment without complications *would be considered to have met the standard for "side effects reported." The research team met frequently, and disagreements were resolved by discussion and consensus. Standardized rating protocols and abstraction forms were used.

### Data analysis

We determined the proportion of all treatment-related case reports adhering to each of the 11 reporting guidelines. The results are presented as percentages with ninety-five percent confidence intervals (95 CIs). After assignment of the initial ratings by the two reviewers, we measured inter-rater agreement using the kappa statistic. Finally, we compared the presence of each of the critical elements among the four journals and across the six years, using chi square statistics.

## Results

### Characteristics of case reports

During the six-year study period ending December 31, 2005 there were 1,316 case reports of all types published in the four peer-reviewed emergency medicine journals. Of these, 85 (6.5%, 95CI = 5.2 – 7.9%) were reports of a treatment. Among the treatment-related case reports, 37 (44%, 95CI = 33 – 54%) described treatments for poisonings or overdoses. There were 52 medical interventions (60%) and 34 surgical or other procedures (40%); one study included both. The majority of reports (69, 81%) included a single case; eight (9%) reported two cases, six (7%) reported 3 cases, and there were two reports with 5 and 6 cases, respectively. The number of treatment-related case reports varied from 5 in 2005 to 21 in 2000. Thirty-one treatment-related case reports were found in the *American Journal of Emergency Medicine*, 29 in the *Journal of Emergency Medicine*, 21 in the *Annals of Emergency Medicine *and four in *Academic Emergency Medicine*. Forty-two percent were reported as letters-to-the-editor.

### Presence of Essential Patient and Treatment Information

Table 2 illustrates the proportion of articles that adhered to the essential reporting criteria. Almost all case reports included the age and gender of the patient. A large majority also presented enough information to enable the reader to understand the nature, stage and severity of the patient's disease, the interventions and the outcomes that were measured. However, critical information was missing in over half of all case reports in each of the following areas: patient medications; co-morbidities; co-interventions; and adverse effects of the intervention. Even smaller percentages alluded to alternative explanations for the favorable outcomes (27%) or to the generalizability of the result (29%). Only 2 case reports included a "denominator" – the number of other patients treated in the same manner, whether successfully or not.

**Table 2:** Proportion of Case Reports Reporting Critical Information

**Critical Element**	**Percent Reporting**	**95% Confidence Interval**
**Description of Patient and Disease**		

Age and gender	99	95 – 100

Definition, severity and stage of disease	88.1	79 – 93

Co-Morbid conditions	45.2	35 – 56

Patient medications	29.7	21 – 40

**Description of Interventions**		

Route or dose of medications or details of procedure described	79.7	70 – 87

Co-interventions	57.1	46 – 67

**Description of Outcomes**		

Clinically relevant outcomes defined	90.4	82 – 95

Side effects reported	33.3	24 – 44

**Threats to Generalizability anad Validity**		

Generalizability	28.6	20 – 39

Alternative explanations	27.4	19 – 38

Information about denominator	2.3	1 – 8

The data were analyzed to determine whether adherence to essential reporting criteria varied across the four journals or over time. There were no significant differences among the four journals, and there were no significant temporal trends.

When we tested for inter-rater agreement for the 11 reporting standards, we found that the kappa values varied from 0.15 to 1.0. All discrepancies were easily resolved by discussion between the senior authors.

### Examples of Reporting Deficiencies

#### Description of patient and disease

A large proportion of case reports did not provide the information needed to confirm the patient's disease or to judge the severity or stage of his condition. For example, one report describing the use of hemodialysis for lithium cardiotoxicity did not report a blood pressure or whether the patient had symptoms of end-organ dysfunction during a bradycardic episode. [22] The reader is left to guess whether the intervention reversed significant cardiotoxicity or simply "treated a number."

#### Description of the intervention

Case reports must include complete information about the treatments the patient received, including medication dosages and routes, important procedures and supportive and adjunctive care. In the current review, errors of omission were common. One published report described the use of warm water immersion to reverse the pain of a lionfish envenomation but failed to state the temperature of the water bath or the duration of immersion. [23]

Incomplete reporting of co-interventions was also common. For example, a case report described the "successful" use of ketorolac for the treatment of chest pain from myocardial infarction. [24] The report did not state whether the patient received aspirin, beta blockers, oxygen or morphine.

#### Description of outcomes

It was common to read that a patient "stabilized within 2 hours," "was discharged in improved condition," "had no further symptoms" or "made a dramatic recovery." In one case report describing the benefits of hemodialysis for a patient who had suffered valproic acid poisoning, we learned only that "the patient's neurologic status promptly improved."[25] The clinician-reader is left wondering: Which symptoms or signs improved? How completely? And for how long?

In the current review, only one-third of case reports informed readers whether side effects were observed. In one case report, a telephone-assisted Heimlich maneuver was "effective" in relieving airway obstruction in a woman who had choked on a piece of meat; however, there was no mention of rib fractures, gastric injury or any other potential complication. [26] In another report, wide-complex atrial fibrillation was "effectively terminated" with ibutalide; however, there was no information about adverse effects, such as QT interval prolongation, hypotension or thromboembolism. [27]

#### Generalizability

It is the authors' responsibility to outline important limitations to the generalizability of their case report. In the case of the telephone-assisted Heimlich maneuver to reverse life-threatening airway obstruction, the authors did not comment on whether the intervention would be equally safe and effective in children, obese patients, the elderly or others. One case report described the use of ultrasound to facilitate aspiration of a breast abscess. The authors wrote, "This convenient bedside technology could make a considerable improvement in patient care,"[28] a conclusion that should be tempered by consideration of the training and experience of the ultrasonographer.

#### Inferences of causality

At the very least, authors of a case report should acknowledge that the treatments administered may, or may not, have caused or contributed to the observed outcome. In a report of the utility of oxygen in myocardial infarction, the authors never acknowledged any of the competing explanations for the resolution of the patient's EKG abnormalities, such as co-interventions (aspirin, beta-adrenergic blockers, nitroglycerine or thrombolytic agents) or the natural history of the patient's condition.). [29] In all treatment-related case reports, the competing explanations – natural history, co-interventions, spontaneous variability and others – should be acknowledged.

#### Absent denominator

In a 2003 case report a single patient with intractable hiccups underwent the Heimlich maneuver with successful termination of his symptoms. [30] The authors should have reported the number of other patients with hiccups who received this intervention by the authors or their colleagues, whether or not the intervention was successful. In another case report, two patients with high fever were treated successfully with intravenous ketorolac. [31] While the authors may be justified in concluding that ketorolac is "effective as an antipyretic," skeptical clinician-readers might ask how many other patients with fever received this intervention, with or without improvement. Reporting only successful outcomes represents a form of publication bias that may mislead readers by implying there was a success rate of 100 percent.

## Discussion

*Some case reports ... eventually prove to be important; most do not. Unfortunately, their methods do not permit discrimination of the valid from the interesting but erroneous, and they cannot provide a sound basis for clinical action*. [32]

Although randomized clinical trials and systematic reviews provide the "best evidence" for guiding practice, some emergency medicine journals still publish case reports. In fact, case reports are over-represented in the literature of emergency medicine, compared with internal medicine or surgery. [11] According to Kidd and Hubbard, editors of the *Journal of Medical Case Reports*, case reports still "have the potential to contribute to and change medical practice.". [6] However, in this study, we found that treatment-related case reports published in four emergency medicine journals often omit critical details about treatments, co-interventions, outcomes, generalizability, causality and denominators. As a result, the information may be misleading to providers, and the clinical applications may be detrimental to patient care.

Case reports serve several purposes. They are a means to disseminate information about novel signs or symptoms of disease and to depict various medical oddities [6,20,33]. They are a critical surveillance tool for rare clinical events [34] And they appear to have a high sensitivity for detecting adverse drug effects and interactions [34,35]. However, there are special hazards when case reports are utilized to guide therapeutics. Over twenty years ago Moses observed that case reports "are most liable to infirmity as an arbiter of treatment effectiveness"[1]

Inherently, treatment-related case reports are limited by the absence of a clear hypothesis, objective or treatment protocol. Their validity is further threatened by small numbers, the absence of a comparison group, vague patient selection criteria, unsystematic observations and unplanned, sequentially or simultaneously administered interventions and co-interventions. Clinician-readers must also confront chance, missing data, short follow-up periods and the conscious or unconscious biases of those who would select the outcomes to report or suggest causal mechanisms. And, as noted earlier, case reports also represent selected "numerators;" while the patient in the report did well, we seldom learn how many other patients were treated, perhaps unsuccessfully.

We acknowledge several limitations to the current study. First, our findings are limited to treatment-related case reports published over a 5-year period in four emergency medicine journals. A large fraction of the case reports were about antidotes for environmental exposures or drug poisonings; only a minority involved a procedure or surgical intervention. Overall, we reviewed a relatively small number of case reports, limiting the precision of the estimates. Second, almost half of the reports we included were letters. As letters may have strict word limitations, it is possible that critical elements were not included or eliminated during editing to meet these space requirements. While we recognize the value of journal space, editors must balance the need to be concise with the importance of adequate case descriptions. Finally, the case reports were judged against 11 selected reporting criteria; other reporting requirements may exist that would further enhance the validity and utility of treatment-related case reports.

This review suggests that case reports frequently omit essential information about the patient, disease, treatment and outcomes. Authors frequently over-interpret their observations. And all too often, authors make treatment recommendations that rely more on deductive reasoning, casual observation, hopeful anticipation and intuition than on valid observations and inferences.

## Abbreviations

CR: case reports; AIDS: Acquired Immune Deficiency Syndrome; MAST: Military Anti-Shock Trousers; 95 CI: ninety-five percent confidence intervals.

## Competing interests

The authors declare that they have no competing interests.

## Authors' contributions

KH and SRL designed the study, reviewed the case reports, and drafted the manuscript. SP and TR assisted in the design of the study, abstracted the case reports and revised the manuscript. All authors reviewed the final draft of the manuscript.

## Pre-publication history

The pre-publication history for this paper can be accessed here:


